# Tools for malaria elimination in the Kingdom of Saudi Arabia

**DOI:** 10.1186/1475-2875-11-S1-O56

**Published:** 2012-10-15

**Authors:** Michael Coleman, Mohammed H Al-Zahrani, Marlize Coleman, Janet Hemingway, Abdiasiis Omar, Adel Al-Shaikh, Ziad A Memish

**Affiliations:** 1 Liverpool School of Tropical Medicine, Pembroke Place, Liverpool, L3 5QA, UK; 2 Ministry of Health, Kingdom of Saudi Arabia

## 

In 1998, the Kingdom of Saudi Arabia (KSA) suffered its worst malaria epidemic. A total of 36,139 locally transmitted cases were recorded and incidence reached as high as 44/1000 in malarious regions, in Southern KSA. Since then, malaria control has been scaled up significantly, with ITNs, larviciding and improved case management. These activities had a significant impact, in 2011 only 29 locally transmitted cases were recorded in the country, reducing incidence to <0.01/1000, well below the WHO recommended rate of 5/1000 that a country should consider before elimination. KSA is now one of the 32 countries that are facing the challenge of malaria elimination.

In order eliminate malaria KSA needs to enhance its surveillance programme, ensuring timely and accurate collection of all malaria operational data. This will be achieved by the integration of passive case detection with reactive and active case detection to rid the country of these last few cases and to prevent malaria from re-establishing itself from imported cases. This data can be used to map, and target, areas of transmission and track any outbreaks. A successful elimination campaign will require the incorporation of other relevant data, for better targeting and faster response to disease detection.

KSA will achieve this by Arabisation of the Malaria Decision Support System, originally developed in collaboration with malaria programmes in southern Africa [1]. This system integrates a number of tools covering case surveillance, intervention planning and monitoring, entomological monitoring and survey development. It also incorporates the ability to generate maps and standard reports at the ‘click of a button’.

One of the key challenges to elimination is detected every case and to respond to that case. The MDSS builds on experiences from Africa [2,3] where simple tools allow for the detection of passive cases, reactive follow up and any action carried out within 48 hours. These tools also allow for the mapping of malaria clusters for better targeting of resources and detection of disease outbreaks, something that will be critical to KSA in avoiding resurgence from imported cases.

Here we demonstrate how the MDSS can be used to track malaria from moderate endemicity to an elimination phase and demonstrate how the system could then be used to assist in elimination of malaria and its maintenance.
